# Soil Chemical Property Changes in Eggplant/Garlic Relay Intercropping Systems under Continuous Cropping

**DOI:** 10.1371/journal.pone.0111040

**Published:** 2014-10-23

**Authors:** Mengyi Wang, Cuinan Wu, Zhihui Cheng, Huanwen Meng, Mengru Zhang, Hongjing Zhang

**Affiliations:** College of Horticulture, Northwest A&F University, Yangling, Shaanxi, China; Agroecological Institute, China

## Abstract

Soil sickness is a critical problem for eggplant (*Solanum melongena* L.) under continuous cropping that affects sustainable eggplant production. Relay intercropping is a significant technique on promoting soil quality, improving eco-environment, and raising output. Field experiments were conducted from September 2010 to November 2012 in northwest China to determine the effects of relay intercropping eggplant with garlic (*Allium sativum* L.) on soil enzyme activities, available nutrient contents, and pH value under a plastic tunnel. Three treatments were in triplicate using randomized block design: eggplant monoculture (CK), eggplant relay intercropping with normal garlic (NG) and eggplant relay intercropping with green garlic (GG). The major results are as follows: (1) the activities of soil invertase, urease, and alkaline phosphatase were generally enhanced in NG and GG treatments; (2) relay intercropping significantly increased the soil available nutrient contents, and they were mostly higher in GG than NG. On April 11, 2011, the eggplant/garlic co-growth stage, the available nitrogen content in GG was 76.30 mg·kg^−1^, significantly higher than 61.95 mg·kg^−1 ^in NG. For available potassium on April 17, 2012, they were 398.48 and 387.97 mg·kg^−1 ^in NG and GG, both were significantly higher than 314.84 mg·kg^−1 ^in CK; (3) the soil pH showed a significantly higher level in NG treatment, but lower in GG treatment compared with CK. For the last samples in 2012, soil pH in NG and GG were 7.70 and 7.46, the highest and lowest one among them; (4) the alkaline phosphatase activity and pH displayed a similar decreasing trend with continuous cropping. These findings indicate that relay intercropping eggplant with garlic could be an ideal farming system to effectively improve soil nutrient content, increase soil fertility, and alleviate soil sickness to some extent. These findings are important in helping to develop sustainable eggplant production.

## Introduction

Eggplant (*Solanum melongena* L.), because of its rich nutritional value, good taste and easily cultivation, is grown in most parts of the world, among which China is the greatest producer [1]. However, in recent years, particularly under continuous cropping in facilities, many problems have affected the sustainable production of eggplant, such as aggravating plant disease and pests, degraded soil physical and chemical characteristics, and declining production and stress resistance of plants [2], [3].

As a simple repetitive agronomic practice, continuous cropping is the practice of growing the same crop year after year in the same field [4]. It makes the soil susceptible to erosion hazard and weed invasion, soilborne pathogens increase, and the survival of certain pathogens enhancing that a certain degree of damage has to be accepted [5]. One of the root causes is that long-term monoculture with a single plant eliminates crop and biological diversity [6]. Consequently, the diversification of crop systems by increasing the number of cultivated species in the same or nearby areas has been proposed to overcome those continuous cropping obstacles. In modern agriculture, crop rotation is the most common for a vast range of crop species worldwide [7]. If properly designed, crop rotation is the most efficient practice to reduce the incidence and severity of soilborne diseases [8]. However, crop rotation is not always practiced because of the difficulty in design of a proper rotation and relatively high risk of losing the lower-value crop. In addition, as the cultivated land is limited and Chinese farmers are accustomed to plant crops of the same species or the same families, it is difficult to carry out crop rotation under protected cultivation in China [9]. Another significant cropping technique - relay intercropping, which is defined as after-crop planting between the rows or plants in later periods of the preceding crops’ growth with a shorter symbiotic time [7], is claimed to promote biodiversity and diversify agricultural outcome compared with monocropping in sustainable agriculture. Intercropping, being looked as an efficient and most economical production system, is drawing more and more attention of small growers [10]. The most common advantage of intercropping is to produce a greater yield and diversified production per unit area and time by achieving higher efficient use of the available growth resources that would not be utilized by each crop grown alone. From the view of diversity restoration, intercropping provides high insurance against crop failure and overall provides greater financial stability for farmers, making the system particularly suitable for labor-intensive small farms or greenhouses [11]. Besides, intercropping offers effective weed suppression [12], [13] and pest and disease control. Crops grown simultaneously enhance the predators and parasites, which in turn prevent the pest build-up, thus minimizing the use of expensive and dangerous chemical insecticides. Mixed crop species can also delay the diseases onset by reducing the spread of disease carrying spores and by modifying environmental conditions so that they are less favorable to the spread of certain pathogens. Moreover, intercropping is an excellent practice for controlling soil erosion and improving soil fertility [11] and quality [14].

Soil sickness, which means serious decline in soil quality, determines the sustainability and productivity of agroecosystems [15]. The changes of the physical, chemical, and biochemical properties of the soil must be taken into account in assessing changes in soil quality [16]. Soil enzyme activities, as mediators and catalysts of most soil transformation processes, have been proposed as appropriate indicators of the health and sustainability of soil quality and ecosystems [17] due to their sensitivity to ecosystem stress [18], intimate relationship with soil biology, and rapid response to changes in soil management [19]. Invertase, widely exists in the soil, plays an important role in increasing the soluble nutrients in the soil [20]. Urease is of great importance in the soil nitrogen cycle and utilization because it can hydrolyze urea to ammonia, one of the sources of plant nitrogen. Another hydrolase, alkaline phosphatase, can mineralize organic phosphorus (P) to inorganic P [21] for plant absorption. Thus, soil enzymes can provide indications of changes in metabolic capacity and nutrient cycling in management practices [22]. Soil nutrients are important factors affecting the growth and development of plants. Nitrogen (N) is the most important element for plant development; it stimulates shoot growth and produces the rich green color that is characteristic of a healthy plant. P is the second most frequently limiting macronutrient for plant growth [23] and plays a major role in the processes requiring a transfer of energy in plants. Another essential nutrient, potassium (K), is a key factor in plant tolerance to stresses such as cold/hot temperatures, drought, and pests. Soil nutrient contents relate to the soil productivity, and soil enzymatic characteristics can reflect the status of key biochemical reactions that participate in the transformation of soil nutrients [24]. However, the reaction rates of soil enzymes are markedly dependent on pH and the presence or absence of inhibitors [25]. In addition, the availability of mineral elements to plants may also be affected by soil pH. Soil pH may affect plant root growth directly or indirectly by impairment of nutrient relations [26]. In turn, growing roots affect the pH of the rhizosphere during plant growth processes and nutrient uptake [27], [28].

In relay intercropping systems, although the increased crop species are expected to overcome the continuous cropping obstacles, the selection of companion crops is still critical. Garlic (*Allium sativum* L.), belonging to the Liliaceae family, is a common vegetable and food spice that is used widely throughout the world. Especially, it is an important economic crop and a good cover crop in vegetable production in China [29]. Garlic products-green garlic, garlic bulbs, and garlic sprouts are all important vegetables favored especially for Asian. It is also commonly used as natural broad-spectrum antibiotic. Khan and Cheng [30] found that garlic root exudates is an effective and environment-friendly management measure against *Phytophthora* blight of pepper and may be used in the organic vegetables production. In addition, it has been reported that the exudates secreted by the rooting system of garlic can produce noteworthy effects on soil structure and ecology [9], [31]. Thus, garlic is expected to be an ideal companion crop for relay intercropping with eggplant.

There is an increasing population and decline in arable land in China, so sustainable agriculture has gained more attention owing to its efficient use of resources, balance with the environment, and the ultimate goal of providing human benefit [32]. In recent years, an increasingly number of studies have focused on intercropping of different grain crops [33]–[35], in addition to intercropping cucumbers [9], peppers [31] or Chinese cabbages [36] with garlic, but eggplant/garlic relay intercropping systems are rarely studied, and intensive study of the soil properties change has been considerably less. For this reason, we have concentrated on comparing the activity of enzymes, content of available nutrients, and pH value in the soil of eggplant/normal garlic or green garlic relay intercropping systems with those in an eggplant monoculture system under continuous cropping to assess if relay intercropping eggplant with normal or green garlic is effective on improving the soil fertility level and maintaining the soil quality, which will help ensure the sustainable long-term development of eggplant cropping.

## Materials and Methods

### Experimental site

**Figure 1:**
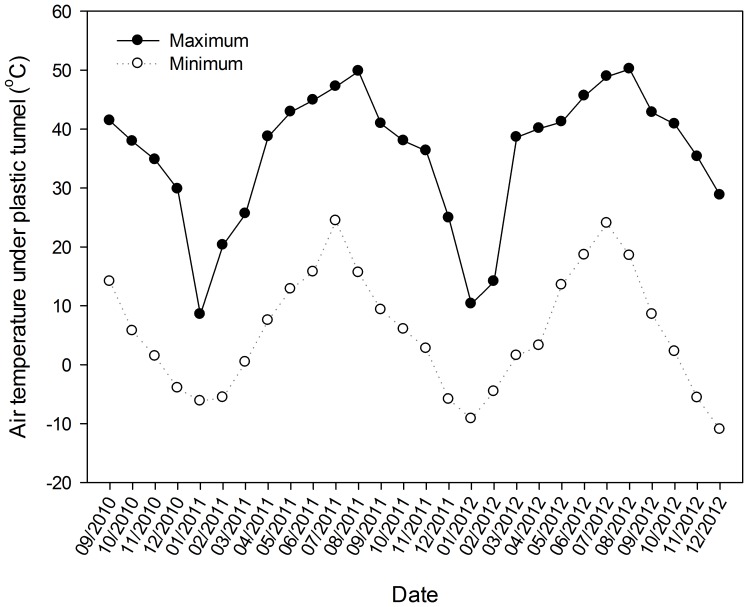
Monthly maximum and minimum air temperature under plastic tunnel in the three experimental years from September 2010 to December 2012. Fig. 1 was drawn using the software program Sigmaplot 12 (Systat Software, Inc.).

The experiment was conducted from September 2010 to November 2012 under a plastic tunnel at the Horticultural Experimental Station (34°17' N, 108°04' E) of Northwest A&F University, Yangling, Shaanxi Province, China, where it is hot in summer and cold in winter, and the annual mean temperature is 12.9°C, with a frost-free period over 200 days. Under plastic tunnel, the highest temperature can achieve around 50°C, and the lowest is around −10°C (Fig. 1).

The soil used for this experiment was brown loamy, alkaline Orthic Anthrosol (Table 1). The soil pH was 7.8 (1∶1 water), and it contained 27.02 g of organic matter, 1.38 g of total N, 0.96 g of total P, and 14.31 g of total K per kilogram dry soil. The ammonium N, available P, and exchangeable K concentrations were 57.17 mg·kg^−1^, 57.65 mg·kg^−1^, and 224.01 mg·kg^−1^ and invertase, urease, and alkaline phosphatase activities were 18.12 glucose mg·g^−1^, 1.99 NH_3_-N mg·g^−1^ and 0.94 P_2_O_5_ mg·g^−1^ respectively in the 0–20 cm soil layer before crop transplanting in March 2010. Cucumber had been planted for approximately ten years before the planting of eggplant in the spring of 2010.

**Table 1:** Basic characteristics of original soil in the experiment.

Soil type	pH value(1soil:1water)	Organicmatter(g·kg^−1^)	Totalnitrogen(g·kg^−1^)	Total phosphorus(g·kg^−1^)	Totalpotassium(g·kg^−1^)	Ammoniumnitrogen(mg·kg^−1^)	Availablephosphorus(mg·kg^−1^)	Exchangeablepotassium(mg·kg^−1^)	Invertaseactivity(glucosemg·g^−1^)	Ureaseactivity(NH_3_-Nmg·g^−1^)	Alkalinephosphataseactivity(P_2_O_5_ mg·g^−1^)	Cultivationhistory
Brown loamy, alkaline Orthic Anthrosol	7.8	27.02	1.38	0.96	14.31	57.17	57.65	224.01	18.12	1.99	0.94	Cucumber planted for ten years

### Experimental design

**Figure 2:**
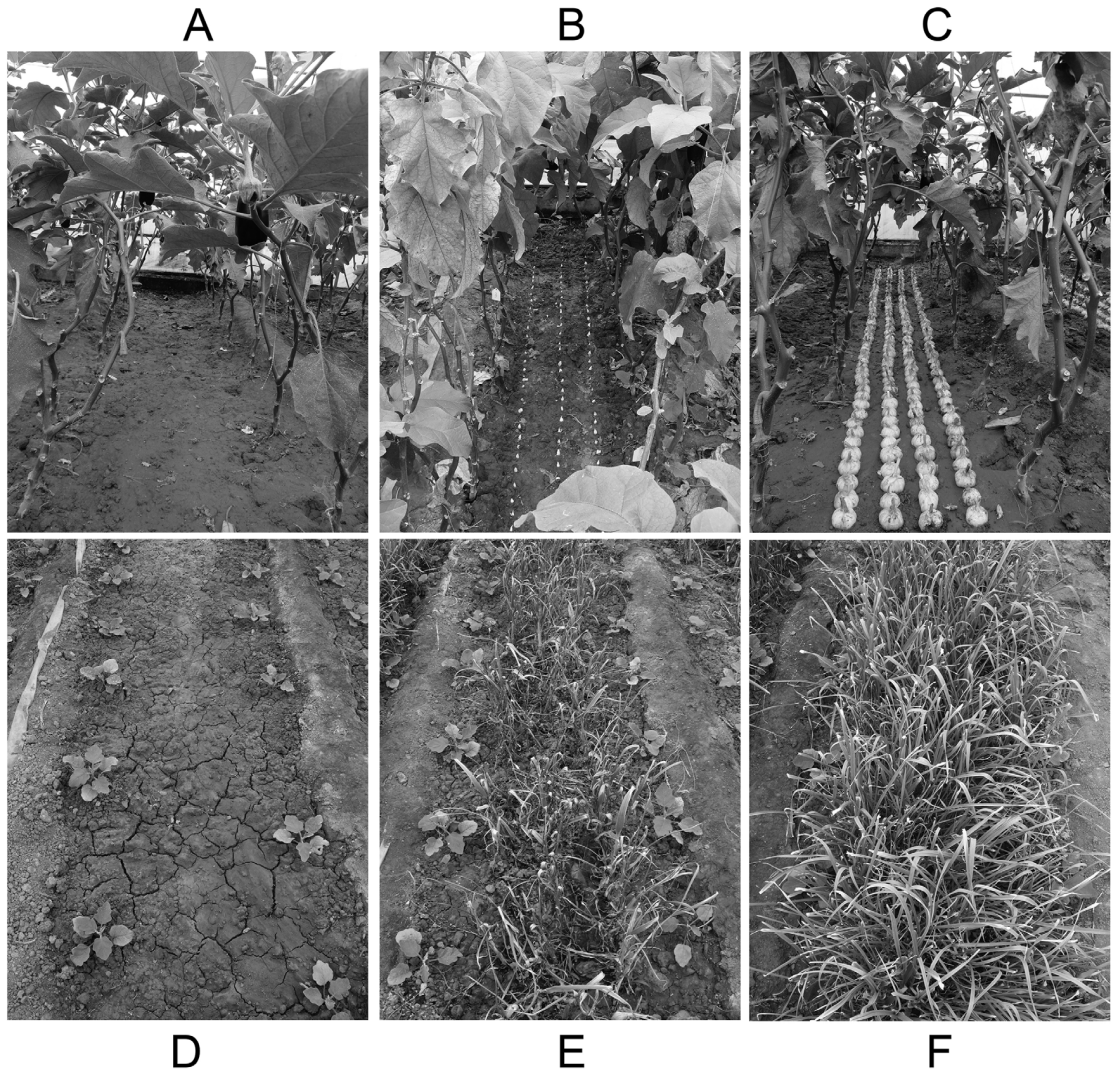
Three experimental treatments in autumn (A–C) and spring (D–F). Fig. 2 was made by graphics software Adobe Photoshop CS6 (Adobe Systems, Inc.). Eggplant monocropping (CK, A and D), eggplant relay intercropping with normal garlic (NG, B and E), and eggplant relay intercropping with green garlic (GG, C and F).

Eggplant as the main crop was relay intercropped with garlic (Fig. 2). A completely randomized design was used consisting of three treatments with three replications: eggplant monoculture (CK) (Fig. 2 A and D), eggplant relay intercropping with normal garlic cv. G110 (NG) (Fig. 2 B and E), and eggplant relay intercropping with green garlic cv. G064 (GG) (Fig. 2 C and F). In NG treatment, normal garlic means garlic bulb, where garlic cloves of uniform size were manually planted into the soil among the eggplant plants in autumn (Fig. 2 B), and expanded garlic bulbs were harvested one by one using shovel in the next spring (Fig. 2 E). In GG treatment, green garlic means green garlic sprouts, where garlic bulbs of uniform size were planted directly by hand in autumn (Fig. 2 C), and the green sprouts were harvested three or four times within the next three months once they were about 20 cm high (Fig. 2 F).

**Figure 3:**
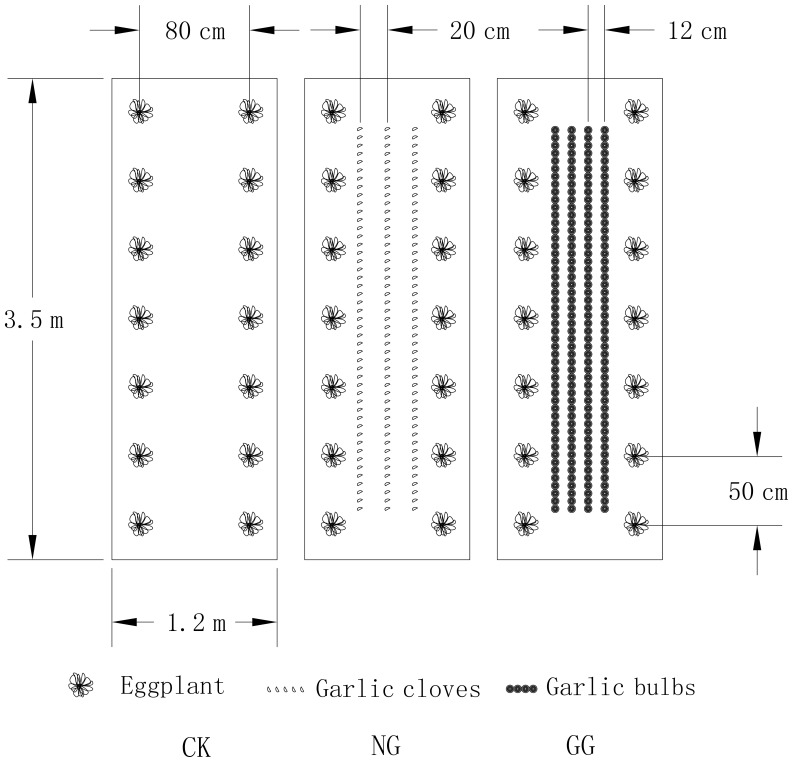
Diagram of three cropping systems in the experiment. Fig. 3 was drawn using the software program AutoCAD 2012 (Autodesk Inc.). CK: eggplant monoculture; NG: eggplant relay intercropping with normal garlic cv. G110; GG: eggplant relay intercropping with green garlic cv. G064.

There were two beds per plot of the three treatments. Each bed was 3.5 m long and 1.2 m wide (Fig. 3). There were two rows of eggplants per bed and seven plants per row, and it was 50 cm for plant spacing and 80 cm for row spacing in both the monoculture and relay intercropping treatments. In the relay intercropping treatments, three rows of garlic cloves (20 cm for row spacing and 6 cm for plant spacing, with 141 cloves for each bed) for NG treatment and four rows of garlic bulbs (10 cm for row spacing and adjacent in each row, with 8.48 kg of bulbs for each bed) for GG treatment were planted in the middle of the bed within the eggplant rows.

Under plastic tunnel, all field operations were performed manually because of the limited land area. Eggplants were manually transplanted on March 19, 2010, March 22, 2011 and March 24, 2012, and uprooted one by one using spade around November 25 in the three years. In 2010, seed cloves and bulbs were planted on September 15, and the three treatments were applied; whereas in 2011 and 2012, the bulbs and seed cloves were planted on August 1/July 20 and September 15, respectively. Every year before eggplant transplantation, the experimental areas were plowed into two shallow furrows and fertilized with 1.5 kg of “PengDiXin” (organic fertilizer, manure substitute; made in Henan Province, China, containing organic matter≥30%, N+P_2_O+K_2_O≥4%, humic acid≥20%, trace element≥2%, and organic sylvite≥5%), 0.15 kg of double superphosphate (chemical fertilizer which can be used to improve alkali soil and supply phosphorus and calcium plant needs; total P≥46%, available P≥44%) and 0.15 kg of “SaKeFu” fertilizer (NPK compound chemical fertilizer; made in SACF, Hebei Province, China, containing total primary nutrient≥40%) per bed as base fertilizer following local farming convention. In the eggplant-only period and eggplant/garlic co-growth period, the same amount of “JinBa” fertilizer (compound chemical fertilizer which is most often used in local vegetable production; made in Beijing, China, containing humic acid ≥3%, trace element ≥6%, N+K_2_O ≥18%, and phosphate and K-solubilizing agent ≥5%) was top dressed on each bed according to the instructions. In the garlic-only stage for NG and GG treatments, only water was administered as required. For eggplant, ving tying, pruning, and other farm management were administered following local convention. No other farm management techniques were needed on garlic (Table 2).

**Table 2:** Field operations in the three years.

Year	Date	CK		NG		GG	
		growth stage	field management	growth stage	field management	growth stage	field management
	03/19		eggplant transplant		eggplant transplant		eggplant transplant
	03/20–09/14			eggplant only	eggplant management		
2010	09/15				plant garlic cloves		plant garlic bulbs
	09/16–11/27	eggplant only	eggplant management	eggplant/garlic co-growth	eggplant management	eggplant/garlic co-growth	eggplant management
	10/01 10/16 11/02 12/07			eggplant/garlic co-growth	eggplant management	eggplant/garlic co-growth	green garlic harvest
	11/28		eggplant uprooted		eggplant uprooted		eggplant uprooted
	11/29–12/31	blank	without any operation	garlic only	garlic watering	garlic only	garlic watering
	01/01–03/21						
	03/22		eggplant transplant		eggplant transplant		eggplant transplant
	03/23–04/14			eggplant/garlic co-growth	eggplant transplant	eggplant/garlic co-growth	eggplant transplant
	04/15				normal garlic (garlic bulbs) harvest		green garlic uprooted
	04/16–07/31			eggplant only	eggplant management	eggplant only	eggplant management
2011	08/01	eggplant only	eggplant management	eggplant only	eggplant management		plant garlic bulbs
	08/02–09/14			eggplant only	eggplant management	eggplant/garlic co-growth	eggplant management
	09/15				plant garlic cloves	eggplant/garlic co-growth	eggplant management
	09/16–11/24			eggplant/garlic co-growth	eggplant management	eggplant/garlic co-growth	eggplant management
	09/15 09/30 10/19			eggplant/garlic co-growth	eggplant management	eggplant/garlic co-growth	green garlic harvest
	11/25		eggplant uprooted		eggplant uprooted		eggplant uprooted
	11/26–12/31	blank	without any operation	garlic only	garlic watering	garlic only	garlic watering
	01/01–03/23						
	03/24		eggplant transplant		eggplant transplant		eggplant transplant
	03/25–04/17			eggplant/garlic co-growth	eggplant management	eggplant/garlic co-growth	eggplant management
	04/18				normal garlic (garlic bulbs) harvest		green garlic uprooted
2012	04/19–07/19			eggplant only	eggplant management	eggplant only	eggplant management
	07/20						plant garlic bulbs
	07/21–09/14	eggplant only	eggplant management				
	09/15				plant garlic cloves	eggplant/garlic co-growth	eggplant management
	09/16–11/27			eggplant/garlic co-growth	eggplant management	eggplant/garlic co-growth	eggplant management
	09/11 09/27 10/21					eggplant/garlic co-growth	harvest green garlic
	11/28		eggplant uprooted		eggplant uprooted		eggplant uprooted

CK: eggplant monoculture; NG: eggplant relay intercropping with normal garlic cv. G110; GG: eggplant relay intercropping with green garlic cv. G064.

Eggplant managements include irrigation, fertilization, pruning, and ving tying. When there was eggplant in the field, water and fertilizer were given only when eggplant needed. During the growth stages, eggplant was pruned to dichotomous branching. The two branches were ving tying when they grew 1 m high between June and July every year. When there was only garlic in the field, garlic managements include only watering.

### Measurements

Soil samples were collected from the plow layer (0–20 cm) in the plots of each treatment (Table 3). Eight soil cores (40 mm in diameter) were removed in a serpentine pattern from the center of two eggplant rows of each bed resulting in 16 soil cores per plot. Subsequently, all sub-samples taken from a single plot were pooled. The first sampling dates per year were nine days before garlic planting in 2010 (September 6) and five days before eggplant transplantation in 2011 (March 17) and 2012 (March 19). In 2010, the other two soil sampling dates were October 16 (eggplant/garlic co-growth period) and November 26 (before eggplant uprooting). Then, in 2011 and 2012, soil samples were taken on April 11/April 17 (eggplant/garlic co-growth period), June 20/June 17 (eggplant full bearing period after all garlic harvested), July 25/July 15 (five days before planting green garlic), August 30/September 10 (fifteen/five days before planting normal garlic), October 10/October 20 (eggplant/garlic co-growth period), and November 20/November 23 (five days before eggplant uprooted) separately.

**Table 3:** Soil sampling dates and corresponding eggplant/garlic growth stages in the three experimental years.

Sampling dates	2010			2011							
				03/1704/1106/2007/2508/3010/1011/20
	09/06	10/16	11/26	2012							
				03/19	04/17	06/17	07/15	09/10		10/20	11/23
Stage	nine days before plantingnormal garlicand green garlic	eggplant/garlicco-growth stage	eggplant/garlicco-growthand before eggplant uproot	five days beforeeggplant transplant	eggplant/garlicco-growth stage	eggplant full bearing periodafter all garlic harvested	five days beforeplanting green garlic	fifteen days beforeplanting normal garlic		eggplant/garlicco-growth stage	eggplant/garlic co-growth and fivedays before eggplant uprooted

### Determination of soil enzyme activity, available nutrient content, and pH value

The soil collected from each treatment was put in a well-ventilated place to air-dry then sieved (1 mm) to analyze the activity of enzymes, content of available nutrients, and pH value in the soil. Determinations of all parameters were performed in triplicate, with values reported as means of each treatment.

The activities of invertase, urease, and alkaline phosphatase in soil were assayed on the basis of the release and quantitative determination of the products of glucose, NH_3_-N and P_2_O_5_; soil samples were incubated with 8% sucrose solution, 10% urea solution or 0.5% disodium phenyl phosphate solution, respectively, in suitable buffer solutions for 24 hours at 37°C, and then, spectrophotometric measurements were performed at 508 nm, 578 nm, and 660 nm, respectively [37].

Alkaline hydrolysis diffusion was used to determine the available N content in soil according to the method of Bermner and Shaw [38] with some modifications. The available N in soil was hydrolyzed by 1.0 mol·L^−1^ NaOH solution to NH_3_, which was absorbed by H_3_BO_3_ indicator solution, and then the NH_3_ absorbed by H_3_BO_3_ was titrated with 0.005 mol·L^−1^ (1/2 H_2_SO_4_) standard acid. Available P was extracted with 0.5 mol·L^−1^ NaHCO_3_ solution, and then the level using molybdenum-antimony-D-isoascorbic-acid-colorimetry (MADAC) at 880 nm by the modified method of Olsen and Dean [39]. Available K was extracted with 1 mol·L^−1^ ammonium acetate neutral solution, and then the level using atomic absorption spectrometry according to the method of Pratt [40] with some modifications.

The soil pH value was determined in a soil:water suspension (1∶1 ratios) with glass electrodes [41].

### Data analyses

Data obtained for each year in this study were analyzed by analysis of variance (ANOVA) using PASW Statistics 18.0 software (IBM, Armonk, New York, USA). The significant differences between the means of the soil enzyme activities, available nutrient contents, and pH values among the monoculture and relay intercropping systems were examined according to Duncan's multiple range test at a P<0.05 level.

## Results

### Effects of relay intercropping eggplant with normal or green garlic on soil enzyme activities

#### Soil invertase activity

**Figure 4:**
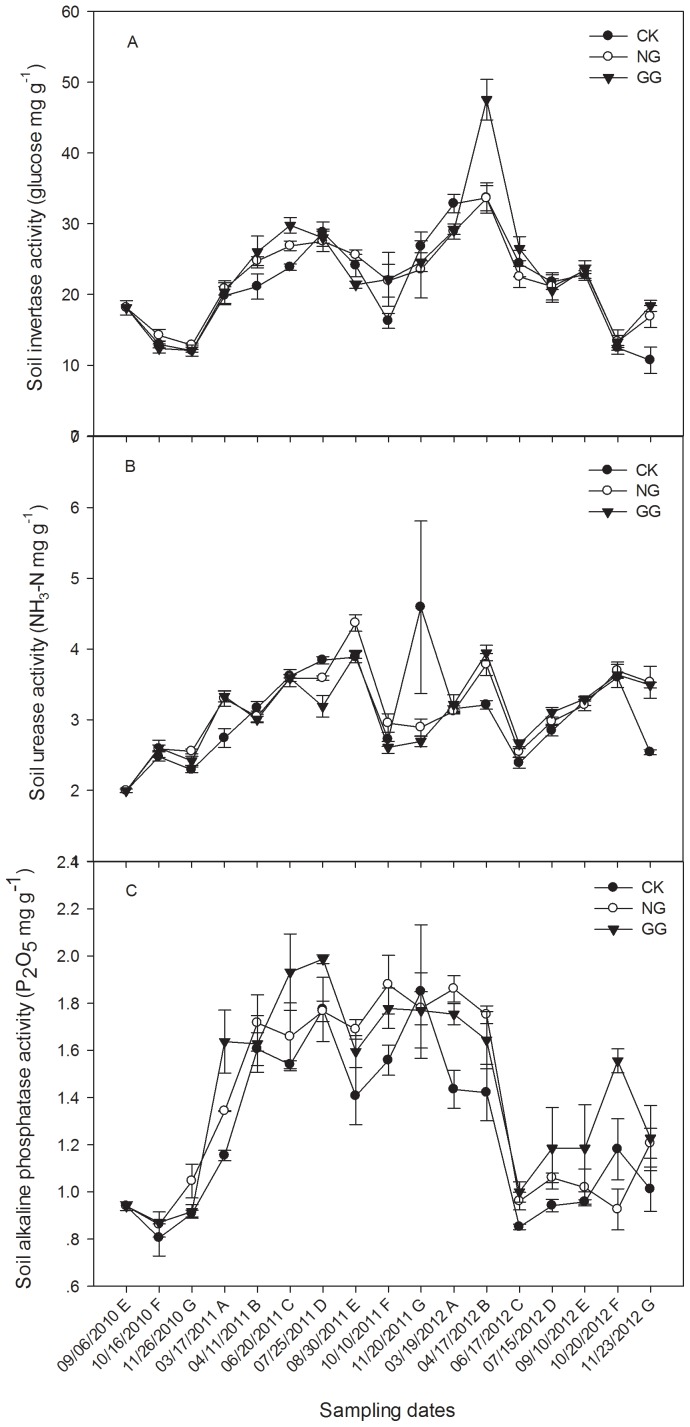
Effects of relay intercropping eggplant with garlic on the activities of invertase (A), urease (B), and alkaline phosphatase (C) in soil from September 2010 to November 2012. Fig. 4 was drawn using the software program Sigmaplot 12 (Systat Software, Inc.). CK: eggplant monoculture; NG: eggplant relay intercropping with normal garlic cv. G110; GG: eggplant relay intercropping with green garlic cv. G064 Error bars represent the standard error of the mean. The capital letters from A to G behind dates in the X-axis represent different periods of crop cycles in the experiment: A represents five days before eggplant transplanted in spring (garlic-only); B represents eggplant/garlic co-growth stage in spring; C represents eggplant-only stage; D represents five days before green garlic planted (eggplant-only); E represents several days before normal garlic planted; F represents eggplant/garlic co-growth stage in autumn; G represents several days before eggplant uprooted (co-growth).

The activities of soil invertase from 2010 to 2012 are shown in Fig. 4 A. In 2010, the invertase activities of all treatments declined as the weather became cold. Then, in 2011 and 2012, the overall trend of invertase activity for all treatments first rose and then fell over time, although there were slight fluctuations in different periods. In 2011, the second experimental year, the soil invertase activity in NG and GG treatments was higher than that in CK treatment during co-growth periods both on April 11 and October 10. On June 20, during the eggplant full bearing period, the invertase activity in NG was 26.87 glucose mg·g^−1^, which was significantly higher than 23.84 glucose mg·g^−1^ in CK. At the same time, it was 29.78 glucose mg·g^−1 ^in GG which was significantly higher than in NG. However on August 30, when the eggplant was relay intercropped with green garlic, but the normal garlic had not been planted, the invertase activity in GG treatment was significantly lower than that in NG treatments. The peak activity appeared on June 20 for the GG treatment and on July 25 for the CK and NG treatments. However, the maximum value of invertase activity of all three treatments appeared earlier in 2012 (on April 17) than that in 2011. On March 19, 2012, the garlic-only stage for NG and GG treatments, the invertase activity in NG and GG treatments showed a significantly lower level than in CK treatment. Then on April 17, during the co-growth period, the activity level in GG treatment was 47.54 glucose mg·g^−1^, significantly higher than that of the CK and NG treatments (33.66 and 33.60 glucose mg·g^−1^). From September 10, 2012, the invertase activity of both NG and GG treatments was higher than that of CK treatment, and the difference even reached a significant level on November 23. In summary, soil invertase activity in relay intercropping treatments was higher than that of the monoculture treatment during the eggplant/garlic co-growth periods, but on other sampling dates, there was no regular routine.

#### Soil urease activity

The activity of soil urease fluctuated in different periods (Fig. 4 B). In the eggplant/garlic co-growth period on October 16, 2010, the urease activity of all three treatments increased over that on September 6 before the garlic was planted. On November 26, though a slight decline, the values of both the NG and GG treatments were still higher than that of the CK treatment, and the difference between NG and CK was significant.

In 2011, the second year of continuous cropping, the overall trend of urease activity continued increasing until it reached the maximum on August 30. On March 17, when there was only garlic in the field, the urease activity of the NG and GG treatments was 3.30 and 3.33 NH_3_-N mg·g^−1^, significantly higher than 2.74 NH_3_-N mg·g^−1 ^in the CK treatment. However, the urease activity of GG was significantly lower than that of CK and NG before the green garlic were planted on July 25. In the subsequent eggplant/green garlic co-growth period on August 30, the urease activity of GG treatment was no longer lower than CK despite the fact that it was still significantly lower than NG, and the activity of the CK treatment was significantly lower than that of NG as well. Later, the urease activity dropped markedly with the decrease of temperature; yet, it presented an abnormal phenomenon for the CK and GG treatment on November 20 that the activity increased again.

Then in 2012, in the third continuous cropping year, the urease activity of the NG and GG treatments was significantly higher than that of the CK treatment during the eggplant/garlic co-growth period, and this positive effect lasted until the green garlic were planted on July 15. However, there was a sharp decrease for all the three treatments on June 17, which may be the result of the application of adequate fertilizer in time during the eggplant vigorous growth stage. On September 10 and October 20, there was no marked difference among the treatments; but on November 23, the urease activity of the relay intercropping treatments was again significantly higher than that of CK.

#### Soil alkaline phosphatase activity

As shown in Fig. 4 C, the overall change trend of the soil alkaline phosphatase activity was similar to urease activity, but varied among treatments and sampling dates. In addition, the alkaline phosphatase activity in 2012 displayed a general decline compared to that in 2011. For the two relay intercropping treatments, the activity was generally higher than that of the CK treatment during the three years of continuous cropping.

In 2010, the alkaline phosphatase activities decreased slightly in the eggplant/garlic co-growth stage on October 16 compared with that on September 6 before the garlic was planted but rebounded before eggplant uprooting. Then in the second year of eggplant continuous cropping, on March 17, 2011 when there was only garlic in the field, the enzyme activity of the GG treatment was 1.64 P_2_O_5_ mg·g^−1^, significantly higher than that of CK (1.15 P_2_O_5_ mg·g^−1^). For the rest of 2011, the three treatments had no significant difference on the alkaline phosphatase activity. In the third year of continuous cropping in 2012, the alkaline phosphatase activity of NG treatment was significantly higher than that of CK treatment on March 19. And for the GG treatment, the activity was always higher than CK, even on March 19 (garlic-only stage), June 17 (eggplant-only stage), and October 20 (eggplant/garlic co-growth stage), the difference reached a significant level.

### Effects of relay intercropping eggplant with normal or green garlic on soil available nutrients

#### Soil available N content

**Figure 5:**
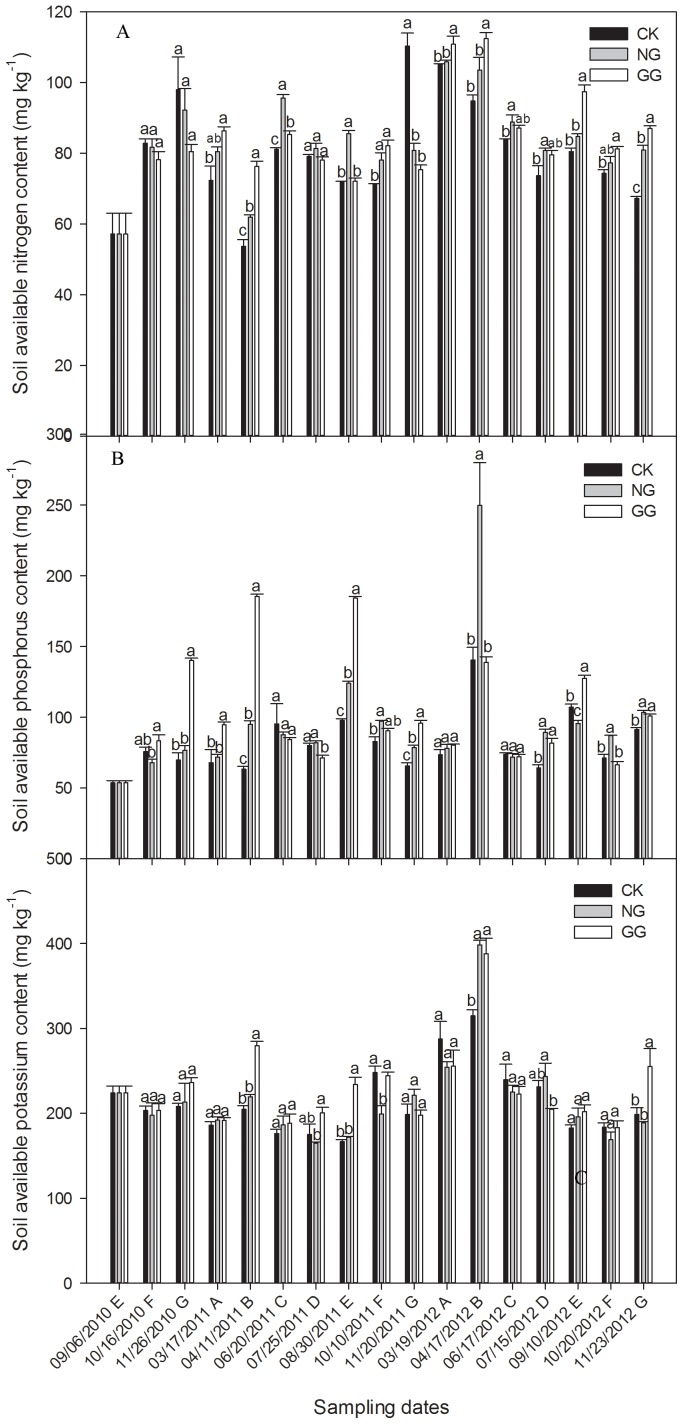
Effects of relay intercropping eggplant with garlic on the contents of available nitrogen (A), available phosphorus (B), and available potassium (C) in soil from September 2010 to November 2012. Fig. 5 was drawn using the software program Sigmaplot 12 (Systat Software, Inc.). CK: eggplant monoculture; NG: eggplant relay intercropping with normal garlic cv. G110; GG: eggplant relay intercropping with green garlic cv. G064 Error bars represent the standard error of the mean. Different letters above the bars indicate significant differences at a P<0.05 level (ANOVA and Duncan’s multiple range test), n = 3. The capital letters from A to G behind dates in the X-axis represent different periods of crop cycles in the experiment: A represents five days before eggplant transplanted in spring (garlic-only); B represents eggplant/garlic co-growth stage in spring; C represents eggplant-only stage; D represents five days before green garlic planted (eggplant-only); E represents several days before normal garlic planted; F represents eggplant/garlic co-growth stage in autumn; G represents several days before eggplant uprooted (co-growth).

Relay intercropping eggplant with garlic affected the content of the main available nutrients in soil. Fig. 5 A shows that, in 2010, the available N content kept rising during the three sampling dates, but there was no significant difference among the three treatments. Then in 2011, the soil available N content of the NG and GG treatments was almost higher than that of the CK treatment, and most reached significant levels. However on November 20, soil available N content in CK was 110.31 mg·kg^−1^, significantly higher than 80.79 mg·kg^−1^ in NG and 75.31 mg·kg^−1^ in GG treatments. In the third continuous cropping year of 2012, the soil available N content of the NG or GG treatments was always significant higher than that of the CK treatment in different periods. The results also indicate that the available N content of the GG treatment was higher than that of the NG treatment in many cases. As a general view, the available N content in the soil of the NG and GG treatments was higher than that in the CK treatment, highlighting a positive effect of eggplant/garlic relay intercropping patterns on increasing the soil available N content.

#### Soil available P content

As shown in Fig. 5 B, the soil available P content of the NG and GG treatments kept increasing over time in 2010 and increased most rapidly for the GG treatment, which was significantly higher than that of the CK treatment on November 26; but for the CK treatment, the available P content first increased and then slightly decreased. In 2011, for the NG treatment, it was always higher than that of the CK treatment, and the difference was significant on most of the sampling dates, but for the GG treatment, the P content was significantly lower than that of the CK before the green garlic were planted on July 25. In addition to that, the available P content in GG treatment was significantly higher than that in CK treatment on many stages, including garlic-only stage (March 17, 2011) and eggplant/garlic co-growth stages (April 11, August 30, and November 20, 2011). Besides, the available P content of the GG treatment was significantly higher than that of the NG treatment on most sampling dates. In the third continuous cropping year in 2012, no fixed change rule was observed among the three treatments. Before the eggplant was transplanted on March 19, the available P content of the three treatments was about the same. Then on April 17, the eggplant/garlic co-growth stage, the P content of the NG treatment was 249.73 mg·kg^−1^, significantly higher than 140.45 mg·kg^−1^ in the CK treatment, but for GG, there was no difference with CK. In the subsequent eggplant-only stage on June 17, when the eggplant grew vigorously, there were no significant differences among the three treatments. On September 10, 2012, when the green garlic had started rooting but the normal garlic had not planted, the available P content of the GG treatment was 127.56 mg·kg−1, significantly higher than 107.24 mg·kg-1 in the CK treatment, but in the NG treatment was 95.73 mg·kg-1, significantly lower than CK. Then on October 20, when the normal garlic grew together with eggplant, the available P content was also significantly higher in the NG treatment than that in the CK treatment. For the last samples in 2012, the available P content in the soil of the NG and GG treatments was significantly higher than that of the CK treatment.

#### Soil available K content

The soil available K content of the relay intercropping treatments on most sampling dates was higher than that of the CK treatment from September 2010 to November 2012 (Fig. 5 C). In 2010 from September to November, the available K content firstly decreased and then increased for all the three treatments. Then in the following spring, decline was seen on the soil available K content on March 17, 2011. In the eggplant/garlic co-growth period on April 11, the available K content of all three treatments increased again compared with that on March 17, and it was 279.52 mg·kg^−1^ in the GG treatment which was significantly higher than 204.49 mg·kg^−1^ in the CK treatment. On July 25 before the garlic was planted, while during the eggplant vigorous growth period, there was no significant difference between the CK and NG or GG treatment. After the green garlic grew up again on August 30, the available K content of the GG treatment was significantly higher than that of the CK treatment. In contrast, the K content was significantly lower than the CK treatment level in the NG treatment after the normal garlic rooting on October 10.

In 2012, the third year of eggplant continuous cropping, the soil available K content in the NG treatment was significantly higher than the CK treatment only during the eggplant/garlic co-growth stage on April 17. For the GG treatment, the K content was significantly higher than that of the CK treatment at two eggplant/garlic co-growth stages on April 17 and November 23.

### Effects of relay intercropping eggplant with normal or green garlic on soil pH

**Figure 6:**
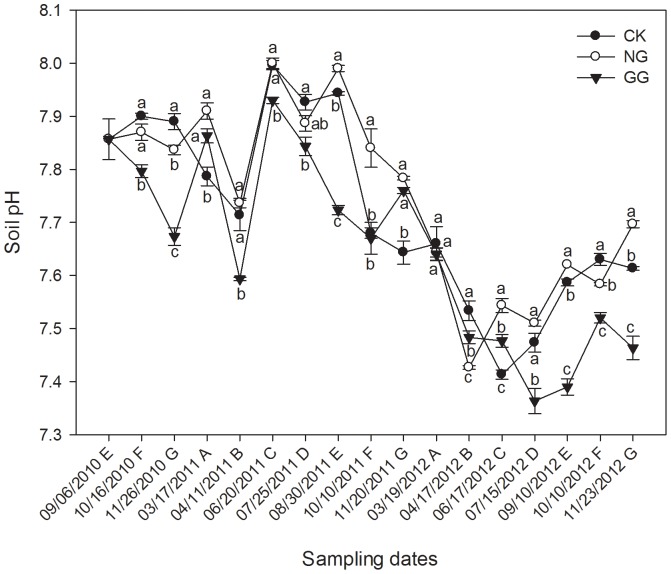
Effects of relay intercropping eggplant with garlic on the soil pH from September 2010 to November 2012. Fig. 6 was drawn using the software program Sigmaplot 12 (Systat Software, Inc.). CK: eggplant monoculture; NG: eggplant relay intercropping with normal garlic cv. G110; GG: eggplant relay intercropping with green garlic cv. G064 Error bars represent the standard error of the mean. Different letters above the bars indicate significant differences at a P<0.05 level (ANOVA and Duncan’s multiple range test), n = 3. The capital letters from A to G behind dates in the X-axis represent different periods of crop cycles in the experiment: A represents five days before eggplant transplanted in spring (garlic-only); B represents eggplant/garlic co-growth stage in spring; C represents eggplant-only stage; D represents five days before green garlic planted (eggplant-only); E represents several days before normal garlic planted; F represents eggplant/garlic co-growth stage in autumn; G represents several days before eggplant uprooted (co-growth).

The soil pH value varied from 7.36 to 8.00 during the three experimental years (Fig. 6). In 2010 from the pre-planting garlic on September 6 to pre-uprooting eggplant on November 26, the soil pH first increased and then decreased in CK and NG treatments, but kept decreasing in GG treatment. Then in both 2011 and 2012, the soil pH of all the three treatments was an initial decrease followed by an increase and a decrease, which might be largely associated with the temperature under plastic tunnel and the water and fertilizer application situation.

On September 6, 2010, the initial value of soil pH was 7.86. Then on October 16, the preliminary growth stage of the normal garlic and green garlic, the soil pH in the GG treatment dropped to 7.80, which was significantly lower than that of CK, might because of a large amount of root exudates from green garlic or the interaction of eggplant and green garlic. However, for the NG treatment, the soil pH decreased unremarkably might because of the lower quantity of garlic. However, by the late eggplant growth period when the garlic thrived, the soil pH of both the NG and GG treatments was significantly below the soil pH value of the CK treatment. Contrary to the results of 2010, in the spring of 2011, when there was only garlic in the fields on March 17, the soil pH in the NG and GG treatments was significantly higher than that in the CK treatment. However, after the eggplant transplanted, the situation changed again. Except on July 25, the soil pH in the NG treatment was higher than CK, and some of the differences were significant. Especially on June 20, soil pH in NG reached up to 8.00. However, contradictory results were found in the GG treatment except on November 20, and the minimum value even dropped to 7.59 on April 11. These results indicate that the soil pH increased in NG with fewer root exudates but decreased in GG with more root exudates. Then in 2012, the results were similar to those in 2011 that soil pH of NG was always higher and in GG was lower in most cases than CK, and the differences were significant on some sampling dates. In addition, the soil pH levels were generally lower in 2012 than that in 2011 in the corresponding period under continuous cropping.

## Discussion

Conventional continuous monocropping may degrade soil quality and negatively affect soil physical processes, and even crop growth potential and yield. Relay intercropping is believed to reduce these negative aspects by maintaining soil quality and it continues to be an important farming practice in developing countries. Higher species richness may be associated with nutrient cycling characteristics that often can regulate soil fertility [42] and limit nutrient losses [43]. Enzyme assays can indicate the situation in terms of soil quality improvement, functional diversity of critical soil processes, rapid responses to changes in management, and sensitivity to environmental stresses [44]–[46]. In turn, soil enzymes are also mainly influenced by vegetation species [21] and land management practices [47]–[49].

Invertase and urease are the most important enzymes in the transformation of carbon and nitrogen in soils [50]. The activity of phosphatase is also positively correlated with the content of soil carbon and nitrogen, and it is also related to the soil pH and organic phosphorus content. Therefore, higher enzyme activities are expected to hold in soil. According to many studies, soil enzyme activities and soil nutrient contents are higher under intercropping systems than under monoculture system [34], [51]. Dai [52] found that intercropping of peanut with *A. lancea* effectively increased soil urease and invertase activities. Li [53] also found that urease activities of intercropping sugarcane and soybean were promoted by 89% and 81%, respectively, compared with that of the monoculture models. In addition, Ahmad et al. [31] found that pepper intercropping with green garlic significantly increased the activities of invertase and alkaline phosphatase in soil. Our results of soil enzymes are in agreement with their conclusions. In our work, the invertase activity in relay intercropping systems was always higher than in eggplant monocropping system during the eggplant/garlic co-growth periods in the three experimental years. For urease and alkaline phosphatase activities, the relay intercropping treatments were higher than those of the CK treatment for most sampling dates. These indicate that the garlic relay intercropped with eggplant stimulated the soil invertase, urease, and alkaline phosphatase activities. In the eggplant/garlic relay intercropping systems, the eggplant and garlic secrete different root exudates, and the root exudates of the two crops interact with each other, affecting the microorganism in the soil, thus increase the soil enzyme activities. It is also possible that the garlic root exudates stimulate the soil enzyme activity by directly acting on them. Exceptional cases appear may result from the influence of many complex factors beyond cropping patterns, such as temperature, fertilizer and water management, or the plant growth situation. In 2011 and 2012, the overall trend of invertase activity of all treatments first increased then decreased over time. This trend could be related to the temperature in the plastic tunnel, which was increasing from March to July then decreasing from August to the end of the year (Fig. 1). In addition, continuous monoculture is detrimental to soil enzyme activities. This was obviously demonstrated by the alkaline phosphatase activity, which displayed a general decrease in 2012, the third year of eggplant continuous cropping, compared with that in 2011. The higher activity of soil alkaline phosphatase in relay intercropping treatments compared with the eggplant monoculture treatment alleviated this decline caused by continuous cropping.

Soil enzyme activity can reflect the level of soil fertility [21], [54]. Increased enzyme activities promote the transformation of soil nutrients and improve the soil fertility. Conversely, soil enzyme activity is also affected by the soil nutrient contents. N, P, and K are the main nutrients in the soil, and plants absorb nutrients in available nutrient form. Previous research has demonstrated that the utilization efficiency of N, P, and K in a maize/mung bean intercropping system were significantly higher than that of a monoculture system [55]. It was also reported by Li [53] that the effective N and P contents of rhizospheric soil of intercrops sugarcane and soybean were increased by 66% and 311.7%, respectively, compared with those in monoculture systems. These results were similar to our work that the available N, P, and K content in relay intercropping treatments were always higher than that in the CK treatment. Urease activity is strongly indicative of enhanced nitrogen transformation in soil [56], [57]. At the initial stage of relay intercropping in 2010, the available N content kept rising, which might be related to the increased urease activity because the urease can promote the replacement of organic nitrogen with available nitrogen. However, the available N content in NG and GG treatments was slightly lower than that in CK, which might because the superiority of relay intercropping was not so obvious at the early stage of relay intercropping, and two crops grown together could absorb more available N than there was only one crop. However, the absorption by garlic did not cause a significant drop in soil available N content and the bad effect is negligible. The results of 2011 and 2012 indicated that the available N content in NG and GG treatments was significantly higher than CK in most stages, but on November 20, 2011, it was significantly lower in NG and GG treatments than CK. This exactly reversed result might because that, the available N was hardly needed for eggplants growing weakly or even dying before being uprooted, but the normal garlic and green garlic were still thriving at that time and needed more available N.

The higher available N, P, and K content in relay intercropping systems compared with that in monoculture systems demonstrates that the root exudates of normal garlic or green garlic stimulated the nutrition availability in soil. In addition, this may be the result of higher enzyme activity stimulated by garlic root exudates increasing the soil available nutrients [58], [59]. Soil enzyme enrichment clearly occurs in response to soil nutrients and vegetation types [21]. This implies that increased enzyme activity is proportionally linked to the improved nutrient cycling and availability. Our study demonstrates that soil enzyme activities and nutrient contents had a similar variation trend in general and relay intercropping eggplant with garlic is better to improve soil fertility. As a result, the external input of N, P, and K chemical fertilizers can be reduced. Furthermore, increased soil fertility leads to good results on crop growth, yield, and land use efficiency. In our study, we also found that the eggplant grew stronger in relay intercropping systems than that in monoculture one, and the eggplant yield and combined output value of per unit area were also slightly higher. Although the eggplant yield declined with the continuous cropping year, relay intercropping could retard the production decrease to ensure the eggplant sustainable production (Data not shown). All these positive results on crop growth and yield could well be related to the higher soil nutrition in relay intercropping systems.

Soil pH is another important property related to soil characteristics and crop growth. Soil pH affects the activity of enzymes and the availability of nutrients [60]. As Acosta-Martínez [61] reported, phosphatase was significantly affected by soil pH, which controlled P availability by the transformation between organic and inorganic P. In other words, the availability of phosphorous in soil depends on the pH. Apparently in our work, the changing patterns of the soil phosphatase activity and pH displayed a similar downward trend in the three years of continuous cropping, which verified that the phosphatase activity was not only harmed by the continuous monoculture but also affected by the decreased soil pH. Results in this study also demonstrated the soil pH in the NG treatment was higher than that in the CK treatment. Increased soil pH led to large increases in nutrient availability [62]. The changes of available N, P, and K content in the CK and NG treatments were the same with soil pH. These results can be explained that the soil urease hydrolyzes urea to form ammonium carbonate, resulting in increased pH [63]. However, for the GG treatment, the soil pH was lower than CK, but the available nutrients contents were still higher than CK. This result was consistent with a study reported that in a wheat/faba bean intercropping system, the rhizosphere pH decreased, but the rhizosphere P availability increased compared with monocropped faba beans and wheat [64]. As is well-known, cropping systems have significant but different effects on soil with time. In relay intercropping systems, the roots of different crops can come into direct or indirect contact, change nutrient conditions and increase interactions, such as competition or mutualism of the two plants [65]. Soil properties based on biological and biochemical activities, especially those involved in energy flow and nutrient cycling, have often been demonstrated to respond to small changes in soil, thus providing sensitive information regarding subtle alterations in soil quality [66]. In eggplant/normal garlic or green garlic relay intercropping systems, both the soil enzyme activities and soil available nutrition content were promoted. However, some results revealed a discrepant change between the two parameters on the same sampling dates. These changes may arise from many intricate aspects of the system that are not yet clear.

Relay intercropping has been shown to motivate higher soil quality and produce more stable yields in a wide range of crop combinations. However, nothing is perfect. We can infer from the results that, the relay intercropping, although being better than monocropping, still exist some problem for improving the sustainability of vegetable production worldwide. Relay intercropping stimulated nutrition more available in soil than monocropping, making crops capture more nutrition, so in the long term, yield advantages of intercropping would have to pay for the higher fertilizer inputs. Besides, mechanization is another problem in intercropping, especially under plastic tunnel with limited land area, intercropping is very labor intensive. However in the developing countries, where manual labor is plentiful and cheap and the work is mainly done by hand in vegetable production, intercropping is still a better cultivation mode.

## Conclusion

Conclusions are drawn from the study that the patterns of eggplant relay intercropping with normal garlic or green garlic can increase soil enzyme activities and available nutrient content and change soil pH, thus improve soil quality and ecological environment. They are expected to help overcome soil sickness and continuous cropping obstacles. It certainly suggests that relay intercropping eggplant with garlic represents a potentially important contribution to meet challenge to sustainable increase the supply of vegetables in China.

Furthermore, it is a reasonable hypothesis that enhanced soil fertility is related to microbial community functions, thus contributing to increased crop growth and yield of the two relay intercropping crops. Clearly, further work is needed to test the microbial community in soil and to elucidate relationships among the soil microorganism, enzyme activity, nutrition, and crop growth and yield. Besides, soil sickness is a result of long term continuous cropping. Longer study periods and larger study plots will help get more convincing results. However, in order to approach local actual vegetable production practice, natural greenhouse environment and field technique used by local farmers need to be kept.
